# Recent Advances in Understanding, Diagnosing, and Treating Hepatitis B Virus Infection

**DOI:** 10.3390/microorganisms8091416

**Published:** 2020-09-15

**Authors:** Magda Rybicka, Krzysztof Piotr Bielawski

**Affiliations:** Department of Molecular Diagnostics, Intercollegiate Faculty of Biotechnology, University of Gdansk and Medical University of Gdansk, Abrahama 58, 80-307 Gdansk, Poland; krzysztof.bielawski@ug.edu.pl

**Keywords:** HBV, HBV RNA, HBcrAg, novel viral markers, direct-acting antivirals

## Abstract

Chronic hepatitis B virus (HBV) infection affects 292 million people worldwide and is associated with a broad range of clinical manifestations including cirrhosis, liver failure, and hepatocellular carcinoma (HCC). Despite the availability of an effective vaccine HBV still causes nearly 900,000 deaths every year. Current treatment options keep HBV under control, but they do not offer a cure as they cannot completely clear HBV from infected hepatocytes. The recent development of reliable cell culture systems allowed for a better understanding of the host and viral mechanisms affecting HBV replication and persistence. Recent advances into the understanding of HBV biology, new potential diagnostic markers of hepatitis B infection, as well as novel antivirals targeting different steps in the HBV replication cycle are summarized in this review article.

## 1. Current Global Status

Almost 60 years after the discovery of Australian antigen (AuAg) and more than 30 years after the approval of the first vaccine, hepatitis B virus (HBV) infection remains the most common chronic infectious disease in humans corresponding to 292 million people globally [1]. According to the World Health Organization (WHO), at least 2 billion people live with serological evidence of past or present infection with HBV, and nearly 900,000 deaths every year are caused by HBV-related complications, including hepatic failure and hepatocellular carcinoma (HCC) [2]. The global incidence of cirrhosis and HCC associated with hepatitis B is around 30% and 45%, respectively, with the proportion as high as 60% and 80% in highly endemic areas (East Asia, Africa) [3]. Importantly, up to 72 million people worldwide are coinfected with hepatitis delta virus (HDV), which is associated with more rapid progression to cirrhosis, a higher incidence of HCC, and increased mortality compared to chronic HBV monoinfection [4].

The epidemiology of HBV infection has always been determined by the detection of the hepatitis B surface antigen (HBsAg) in the general population. In concordance to these data the geographic prevalence of HBV infection is classified into three regions of high (>8%, East Asia, Africa), medium (2–8%, Mediterranean, Eastern Europe), and low (<2%, North America, Western Europe) endemicity [1]. Over the past few years, the epidemiology of HBV infection is changing due to the introduction of the universal vaccination program, hepatitis B screening programs as well as through the human migration between high and low-risk areas [5]. Currently, the complete vaccination schedule in healthy individuals consists of three doses of vaccine and according to the clinical trials the first dose should be delivered as soon as possible after birth (within 24 h) to prevent perinatal HBV infection and induce immunity against HBV. Despite the WHO recommendations, by 2015 only 55% of European countries reported offering Hepatitis B Birth dose (HepB-BD) as part of their National Vaccine Plan, while in remaining countries infants received the first dose of vaccine at 2 months of age or even later [6]. Therefore, the current main route of HBV is vertical, or mother to child transmission (MTCT) that is responsible for approximately 50% of the global disease burden [7]. Nevertheless, other sources such as sexual contact, inadequate sterilization of health care instruments, administration of contaminated blood products, as well as intravenous drug use also remain important modes of transmission. Moreover, special attention should be paid to highly endemic areas (Asia Pacific, sub-Saharan African) where the vertical transmission remains predominant route of infection. The majority of HBV carriers in these regions are infected at birth or during their first 5 years of life, when the risk of progression to chronicity is high [8,9,10]. This phenomenon is caused by the low coverage of the HBV birth dose, concerns about appropriate vaccine storage, unsuccessful prophylaxis against HBV, as well as high incidence of home births [8,10]. Another reason for HBV infection spreading is the poor or non-response to HBV vaccination. Despite the overall high efficiency of the vaccine, still, around 5% of individuals do not respond to the primary HBV series and the cause of this phenomenon remains unclear [11,12]. What should be also considered is the fact that the undetermined group of patients infected with HBV remains underdiagnosed by routine serological tests. These are individuals infected with escape mutants that lack HBsAg, or carriers with an occult infection which is defined as the persistence of viral genetic material in the liver in the absence of HBsAg in serum. According to the Global Hepatitis Report from 2017 only 9% of 257 million patients with a chronic infection have been diagnosed and knew of their status and in consequence as low as 1% of HBV carriers are properly treated worldwide. Furthermore, around 25% (15–40%) of those with chronic hepatitis B (CHB) if left untreated would develop cirrhosis, liver failure, or hepatocellular carcinoma (HCC). All these facts together with the lack of curative therapy against HBV were the cause of WHO implementation of the first global strategy to eliminate HBV infection as a public health threat by 2030 [13].

## 2. HBV Virology

The human HBV which belongs to the family *Hepadnaviridae* has one of the smallest viral genomes with a high organization in the enveloped viral particle. The circular, partially double-stranded DNA genome of HBV (relax circular DNA, rcDNA) is about 3.2 kb in size and comprises a complete coding minus strand (−) and a shorter noncoding plus strand (+) with a fixed 5′ end and a variable length 3′ end [14]. The minus strand of HBV encodes for highly overlapping four long open reading frames (ORFs: preC/C, P, preS/S, and X) that give rise to five RNA transcripts and seven viral proteins which include DNA polymerase (Pol), two nucleocapsid core proteins: hepatitis B core antigen (HBcAg) and hepatitis B e antigen (HBeAg), X protein (HBx), and three envelope proteins: large (L-), middle (M-), and small (S-) hepatitis B surface antigen (HBsAg) [15]. Unlike other DNA viruses, HBV has evolved an unusual genome replication strategy that allows for active replication without destroying the infected cells. Namely, the virus replicates through reverse transcription of an RNA intermediate, the pregenomic RNA (pgRNA), using the reverse-transcriptase activity of the viral polymerase which lacks the proofreading activity. Due to its replicative strategy and the complex nature of the genome, HBV exhibits an estimated 10-fold higher mutation rate than other DNA viruses [16]. Particularly, *P* gene overlaps with all other coding regions and any mutation in the polymerase gene may also affect the overlapping *S* gene influencing viral infectivity, liver disease severity, and also a response to antiviral treatment [14].

### HBV cccDNA

**Figure 1:**
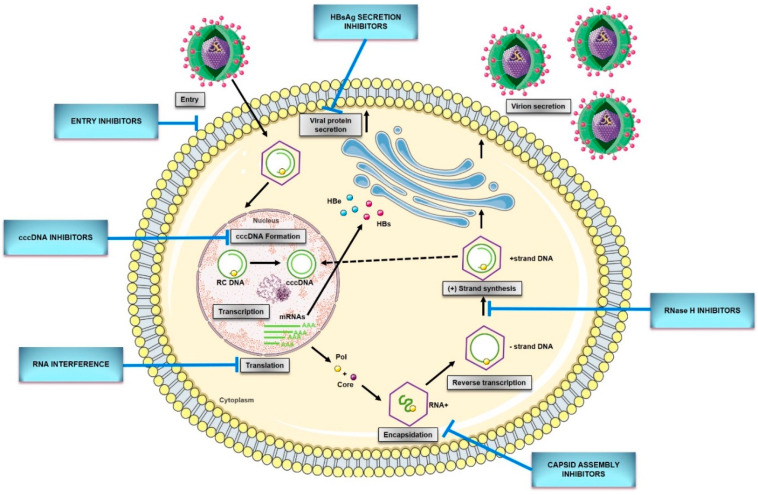
HBV replication cycle and new direct-acting antivirals for HBV. **Entry:** the replication cycle begins with the reversible binding of S-HBsAg to the glypican 5 (GPC5) protein [17,18], followed by specific binding between preS1 region of L-HBsAg and HBV entrance receptor—Sodium Taurocholate Cotransporting Peptide (NTCP) [19]. Upon the entrance, capsids are transported within the cytosol to the nucleus during which uncoating and release of the viral genome are initiated [14]. **cccDNA formation**: the molecular basis of rcDNA to cccDNA conversion remains unclear but it requires (1) the release of the viral polymerase covalently attached to the minus DNA strand by tyrosyl-DNA-phosphodiesterase 2 (TDP2) or its related proteins [20]; (2) the removal of the RNA primer from plus DNA strand by unrecognized enzymes; (3) cleavage of terminally redundant sequences (r) from the negative strand by structure-specific endonuclease 1 (FEN1) activity [21]; (4) the competition of positive DNA strand by the cellular replicative machinery: DNA polymerase κ [22] or polymeraseα, δ, and ε [23], and DNA topoisomerase I and II [24]; (5) the ligation of both viral DNA strands by DNA ligase LIG I and LIG III [25]; (6) chromatinization involving histone chaperones, chromatin remodelers, transcription factors, and viral proteins [26]. **Transcription and translation:** cccDNA transcribes into all viral RNAs necessary for protein production and viral replication utilizing the cellular transcriptional machinery: 3.5-kb pregenomic RNA (pgRNA)/preC RNA and 2.4-kb, 2.1-kb, and 0.7-kb subgenomic RNA [15]. The pregenomic RNA encodes both the polymerase and core protein and works as a template for viral DNA replication. The three subgenomic RNAs encode envelope preS1 protein, preS2, and HBsAg proteins and the X protein [27]. The precore mRNA is translated into precore protein, which is processed at the N-terminal and C-terminal ends to HBeAg, a secretory protein. Pregenomic RNA is reverse transcribed into HBV DNA and also translated into core protein (HBcAg), which overlaps with HBeAg [28]. **Encapsidation:** the first step of HBV genome replication is the encapsidation of the pgRNA by core protein, forming an immature nucleocapsid. This process requires the cis-acting packaging signal (a stem-loop structure termed as epsilon, ‘ε’) at the 5′ end of pgRNA, and phosphorylation of C-terminal of the core protein [15]. **Reverse transcription:** tyrosine residue hydroxyl group of polymerase protein covalently binds at the ε region of 5′ pgRNA initiating the reverse transcription [14]. Next, the first three nucleotides from the bulge region of ε stem-loop are synthesized, and the polymerase with the covalently attached trinucleotide sequence translocates from ε to direct repeat, DR1 located at the 3′-end of the 3.5-kb pregenomic RNA. Following the minus-strand elongation, pgRNA template is degraded by an RNase H encoded within the pol protein [29]. **Pus-strand DNA synthesis**: the terminal 16–18 nts from the 5′ end of pgRNA remains uncleaved and serves as the primer for plus-strand DNA synthesis after it translocates to a complementary sequence on the minus-strand template [30]. Circularization then occurs, facilitated by the short terminal redundancy on the minus strand. Elongation and completion of plus-strand DNA synthesis yield a relaxed-circular DNA genome [14]. **Virions secretion:** HBV genome is packaged into an icosahedral capsid composed of the HBV core protein (HBc), termed nucleocapsid. RC DNA-containing mature nucleocapsids are enveloped by HBV envelope glycoproteins proteins through the ESCRT machinery in the Golgi and secreted extracellularly as complete virions. A portion of capsids are transported back to the nucleus to maintain the pool of cccDNA [31]. cccDNA, covalently closed circular DNA; pgRNA, pregenomic RNA; RC DNA, relaxed circular DNA; HBeAg, hepatitis B e antigen; HBsAg, hepatitis B surface antigen; mRNA, messenger RNA; Pol, HBV polymerase ; Core, HBV core protein.

The replication cycle of HBV (Figure 1) begins with the reversible binding of the small envelope protein (S-HBsAg) to heparan sulfate proteoglycans (HSPG) on the hepatocyte membrane (Figure 1) [17]. Recently, Verrier et al. [18] demonstrated that glypican-5 (GPC5), a protein associated with proteoglycans, functions as an attachment factor during the entry of HBV. Following the proteoglycan binding, a specific HBV entrance receptor, Sodium Taurocholate Cotransporting Peptide (NTCP), is recognized by the preS1 region of HBV large envelope protein (L-HBsAg) and it is the N-terminal myristoylated peptide corresponding to amino acids (aa) 2–48 of the pre-S1 that binds to NTCP with high affinity [19]. Upon entrance, capsids are transported within the cytosol to the nucleus during which uncoating and release of the viral genome is initiated. Once the rcDNA enters the nucleus, it undergoes processing to be converted into viral replication intermediates, (covalently closed circular DNA, cccDNA), which is the template for transcription of all viral RNAs [14,15]. The cccDNA is a stable nuclear form of the viral genome representing the molecular persistent reservoir of HBV. The cccDNA exists in the hepatocyte nuclei as a minichromosome that can be modified by host histone proteins (H3, H4) and non-histone viral and cellular proteins (HBx, HBc, host epigenetics-related proteins). The cccDNA pool is highly stable in hepatocyte nuclei because cccDNA structure is hard to eliminate, and infection of new hepatocytes by progeny virions can be continuous [32,33]. Although HBV cccDNA lifetime is still unknown, it can persist throughout the natural lifespan of hepatocytes. Therefore, reactivation of viral replication from persistent cccDNA is the principal source of recurrence of clinical hepatitis in patients after stopping antiviral therapy, or after being immunocompromised due to chemotherapy, organ transplantation, immunosuppressive therapy, or coinfection with HIV. It has been established that only a few cccDNA copies per liver can reactivate full virus production. Hence, any cure of CHB requires the elimination of cccDNA or, at least, permanent silence to achieve a functional cure that is sustained undetectable HBV surface antigen (HBsAg) and HBV DNA levels in serum, with or without the appearance of antibodies to the HBsAg (anti-HBs) [29,34].

Although many aspects of the HBV replication cycle are well characterized, the molecular basis of rcDNA to cccDNA conversion remains unclear, except that the multiple complex steps are necessary. Formation of cccDNA requires multiple enzymatic reactions for the release of the viral polymerase covalently attached to the minus DNA strand, the removal of the RNA primer from the plus DNA strand, the competition of positive-strand, as well as the ligation of the two viral DNA strands [29]. Considering the small viral genome, all the activities, or at least most probably come from the host cellular DNA damage response. In 2014, tyrosyl DNA phosphodiesterase-2 (TDP2) was identified as the first host DNA-repair factor involved in the formation of HBV cccDNA. Recent studies have demonstrated that TDP2 can release the RT from the 5′ end of minus-strand DNA in vitro [20]. A few years later, the flap endonuclease 1 (FEN1) has been reported to remove the 5′-flap structures formed by RNA oligomer and the redundant fragment (r sequence) [21]. Following the removal of flap structures in rcDNA, the plus-strand DNA must be next extended by HBV polymerase and few host cellular polymerases. DNA polymerase κ (POLK) was reported as a key cellular factor involved in cccDNA formation, which supports HBV infection. However, DNA polymerase L (POLL) and H (POLH) were also demonstrated to participate in this process [22]. Recent studies have revealed that both linear strands are ligated by host DNA ligases: LIG I and LIG III. Additionally, LIG IV was also considered to play a role in forming defective cccDNA from the double-stranded linear (dslDNA) through nonhomologous end joining (NHEJ) DNA repair pathway [22,35].

Despite huge progress in the study of cccDNA that has been made recently, there are many unsolved issues left which might be crucial for developing strategies for a cure of chronic hepatitis B. Thus, further basic studies need to determine other host factors involved in cccDNA synthesis which could help in a deeper understanding of cccDNA formation mechanisms. Moreover, since the amount and transcriptional activity of cccDNA is important for evaluating disease activity and treatment response, it is urgently important to identify novel serum markers that could reflect intrahepatic cccDNA contents and activity.

## 3. Novel Viral Markers of Hepatitis B

### 3.1. HBV RNA

Research works show that circulating HBV RNA can be found in the blood of patients with chronic and acute HBV infection. Both, the nature and the biological role of encapsidated RNA, has not been explained so far, but it is most likely a 3.5 kb genomic RNA (pgRNA) that comes from putative pgRNA-containing virus particles that are a part of the HBV replication cycle [36]. Although it is still unclear how pgRNA is released into the circulation, its presence in sera has recently been reported as the potential marker for HBV-associated diseases. Serum pgRNA becomes detectable shortly after HBV DNA and its level correlates with the viral load [37]. In the absence of treatment HBV RNA is approximately 10^6^ copies/mL which corresponds to ≈2 log less than HBV DNA [38].

Lately, a growing number of studies have inferred that serum HBV RNA can be a useful marker for monitoring the efficacy of antiviral therapy [39,40]. For nucleos(t)ide analogues (NAs) therapy it was shown that the decline in serum HBV RNA levels at 12 weeks of treatment independently predicts both the initial virological response and HBeAg seroconversion [41]. According to these findings, the association was found between the HBV RNA present at the time of NAs treatment discontinuation and the viral rebound. Undetectable HBV RNA was proposed as the marker for NAs therapy discontinuation [36,42,43]. The ability of serum HBV RNA to predict the long-term outcome of NAs treatment was demonstrated in 2020 by a post-hoc analysis of data from a multi-center randomized controlled trial. Fan et al. [44] have shown that individuals who were negative for HBV RNA at the end of treatment, had a significantly lower risk of hepatitis relapse. The authors have also proposed to use the total HBV nucleic acid level (both viral DNA and RNA) as a reliable biomarker for NAs cessation. Due to the completely different mechanism of action, pegylated interferon α (Peg-IFNα) is expected to have a much greater effect on the declining HBV RNA level. While NAs inhibit the reverse transcription of HBV and the formation of new virions, they do not disable cccDNA so that the production of HBV RNA remains unperturbed [36]. Peg-IFNα induces cccDNA degradation and therefore causes a reduction in HBV RNA levels [45]. Several studies have already demonstrated the kinetics of serum HBV RNA is associated with the serologic response following treatment with Peg-IFNα. Different cut off values of HBV RNA level was reported as the negative predictive value for treatment response. Nevertheless, all results indicate a profound decrease in serum HBV RNA during Peg-IFNα treatment and the authors emphasize serum HBV RNA as an early predictor for sustained immune control following treatment with Peg-IFNα [39,43,46,47].

Serum HBV RNA should be also considered as a potential marker for the functional cure of chronic hepatitis B. As HBV RNA is a direct transcriptional product of cccDNA it should be consistent with the intrahepatic activity/amount of cccDNA molecules persisting in HBV-infected patients. However, literature show rather no quantitative correlation between serum HBV RNA and intrahepatic cccDNA load during both NAs and Peg-IFNα therapies [39,48,49,50]. Despite the above data, serum HBV RNA levels appeared to be strongly correlated with intrahepatic HBV pgRNA, as well as with the intrahepatic ratio of HBV pgRNA to cccDNA. Although HBV RNA is not associated with cccDNA it correlates strongly with the liver injury and may reflect the activity of cccDNA [50]. Not only have the serum HBV RNA levels been associated with a degree of intrahepatic inflammation, but also the presence of pgRNA can induce the production of interferons and inflammatory cytokines in hepatocytes that cause histological changes [51]. Thus, circulating HBV RNA has been proposed as a useful biomarker for predicting the occurrence of HCC [52]. Although serum HBV pgRNA may act as a clinical marker for cccDNA activity, HBV RNA levels may differ across the natural course of chronic HBV infection as well as between untreated and treated patients [53,54].

While growing evidence supports that the serum HBV RNA may be used as a new biomarker for HBV infection, treatment, and even prognosis (Table 1), there are still many questions that should be answered. First of all, the molecular biology of serum HBV RNA should be understood together with uncovering the mechanism by which viral RNA facilitates HBV replication. Secondly, because the species and forms of serum HBV RNA have not been rigorously characterized in all studies, the accurate and standardized protocols for serum HBV RNA detection and quantitation is hardly needed.

### 3.2. HBcrAg

Hepatitis B core-related antigen (HBcrAg) consists of three related viral proteins that share an identical 149 amino acid sequence. These components include HBV core antigen (HBcAg), HBV e antigen (HBeAg), and a 22 kDa precore protein (p22Cr) which can be detected and measured altogether in serum by HBcrAg assay that utilizes the chemiluminescence method [78]. To date, several studies have reported that HBcrAg concentration strongly correlates with the HBV DNA levels regardless of HBeAg status [79,80].

However, two independent studies have demonstrated that serum HBcrAg levels may vary within different phases of HBV infection [60,81]. In both studies, serum HBcrAg level was significantly higher in HBeAg-positive patients compared to HBeAg-negative patients without antiviral treatment. Nevertheless, the overall correlation with HBV DNA levels remained significant throughout phases of the disease. More importantly, serum HBcrAg was proposed as a valuable marker to distinguish HBeAg-negative patients with active and inactive disease. Seto et al. [60] have also demonstrated a strong correlation between HBcrAg and highly sensitive quantitative HBsAg levels (HQ-HBsAg). Apart from the antigen-antibody complexes that are detected in the conventional HBsAg assay, HQ-HBsAg detects also HBsAg proteins.

Serum HBcrAg may also serve as a valuable marker for viral replication because it reflects the transcriptional activity of intrahepatic cccDNA [68,69]. As was demonstrated by Testoni et al. [69], HBcrAg levels are associated not only with intrahepatic HBV DNA and cccDNA but also with pgRNA in both HBeAg-positive and HBeAg-negative patients. They have proposed a cut off value for HBcrAg (<3 log U/mL) for identifying inactive carriers, which together with HBV DNA ≤ 2000 IU/mL seems to be more precise as it worked accurately in identifying inactive carriers, regardless of HBV genotype. Another interesting finding was presented in the study of paired liver tissues and serum of patients treated with NAs. Namely, when a serum concentration of HBV DNA decreased to an undetectable level, HBcrAg was still present in 78% of patients and correlated well with cccDNA. Therefore, it may be useful for identifying patients with transcriptionally active cccDNA when viral DNA level decreased below the detection limit [82].

Recently, attention has been paid to HBcrAg as a prognostic factor of spontaneous seroconversion of both HBeAg and HBsAg. Two independent studies have revealed that serum HBcrAg levels were associated with spontaneous loss of HBeAg in chronic hepatitis B patients [58,61]. In a Japanese study, HBcrAg is predictive of early spontaneous HBeAg seroconversion at 12 months [58]. In another study baseline and HBcrAg levels at week 28 of follow-up were positive predictive values for HBeAg seroclearance [61]. The amount of HBcrAg was associated with the clinical profile of the disease in HBeAg-negative chronic hepatitis. Significantly higher HBcrAg levels were found in the HBeAg-negative active phase which was correlated with more advanced necroinflammatory activity and significant fibrosis in comparison to HBeAg-negative inactive carrier phase [83,84]. Nevertheless, the predictive value of HBcrAg levels for HBsAg needs further examination. However, it has already been demonstrated that the majority of patients with spontaneous HBsAg seroclearance have undetectable HBcrAg levels (79%). Moreover, the median value in those who had detectable HBcrAg was relatively low [59,60,62].

The HBcrAg kinetics has been proposed as a treatment predictor in patients undergoing antiviral treatment. The lower level of HBcrAg at the time of HBeAg seroclearance was associated with the likelihood of recurrence in patients treated with NAs and HBcrAg was detectable even when HBsAg was lost [55]. Additionally, a linear correlation between baseline serum HBcrAg or its decreased value and intrahepatic cccDNA has been demonstrated for NAs-experienced patients [85]. Monitoring of HBcrAg levels has been demonstrated as a treatment predictor for Peg-IFNα in patients with both HBeAg-positive and HBeAg-negative chronic hepatitis B. Again, HBcrAg was indicated as a surrogate marker of intrahepatic cccDNA levels and its transcriptional activity. Moreover, together with HBsAg, HBcrAg was proposed as a promising tool for identifying patients with a low probability of persistent virologic remission and HBsAg clearance in a long-term follow-up [56,57].

The incidence of HBV reactivation associated with immunosuppressive therapy may also be predicted by serum HBcrAg measurement in occult viral carriers. A recent study of 124 oncohematological individuals with serologically resolved HBV infection (HBsAg–/anti-HBc+) and undetectable HBV DNA has revealed a 3-fold increased risk of HBV reactivation in patients who had detectable HBcrAg before treatment [63].

Serum HBcrAg was shown to predict hepatocellular carcinoma development. In a large cohort study of non-treated patients, the level of HBcrAg was associated with a 5-fold increased risk of HCC. Additionally, the predictive power for HCC development was superior for HBcrAg in comparison to HBV DNA [64]. Another study confirmed the utility of HBcrAg as a good prognostic predictor for HCC patients and proposed serum HBcrAg as an acceptable method to assess viral load and to monitor cccDNA regardless of HBeAg status [66]. For treatment-experienced patients, the presence of HBcrAg after at least 2 years of NAs treatment was an independent risk factor for HCC development [86]. Furthermore, HBcrAg levels before treatment with NAs were significantly higher in patients who developed HCC in comparison to the control group [65]. The next investigation, which combined HBcrAg and HBsAg levels, has presented that low HBsAg and high HBcrAg levels predispose patients to HCC occurrence [87]. On the other hand, some findings showed the predictive value of HBcrAg in HCC recurrence after curative surgical treatment. Higher levels at the time of HCC diagnosis were associated with a higher probability of HCC recurrence [80]. A more recent report has shown that HBcrAg is a prognostic predictor of HCC and a valuable marker of cccDNA levels especially in patients with low viral loads [66].

Despite the number of reports regarding HBcrAg as an emerging marker for chronic hepatitis B virus infection (Table 1), the interpretation of data should be treated with caution. First of all, most of the studies were conducted in Asian countries and large cohort studies are still lacking for US, European, and Sub-Saharan African countries. Secondly, the measurement of serum HBcrAg requires more validation studies accounting for the HBsAg status, the phase of the disease, and duration of antiviral treatment to identify specific cut-off values for each clinical outcome. Once performed it can be used in clinical practice as a very promising non-invasive HBV marker.

### 3.3. Anti-HBs and Anti-HBc Antibody Titers

Quantification of anti-HBs and/or anti-HBc has been recently investigated in the context of reactivation of HBV infection in patients undergoing high-risk immunosuppressive therapy. In 2014, Seto et al. [70] reported for the first time that undetectable anti-HBs at baseline may be a predictive factor in patients with resolved HBV infection receiving chemotherapy. These findings were confirmed in a multivariate analysis, were anti-HBs negative at baseline, and levels below 100 mIU/mL were significantly correlated with the risk of HBV reactivation [71]. Similar cut-off values were demonstrated in a group of 336 patients undergoing kidney transplantation and in a retrospective study including 108 resolved hepatitis B patients [72,74]. In line with these results, a large meta-analysis of 1672 patients under immunosuppressive therapy revealed anti-HBs positivity as a decreased risk of HBV reactivation underlying the importance of investigation of the anti-HBs levels [73].

Quantification of anti-HBc antibodies (whether IgM or IgG) may help distinguish prior HBV infection from occult or ongoing HBV infection because their level changes depending on the stage of HBV infection [77]. Anti-HBc titer may also play a role in identifying patients with a high risk of HBV reactivation. According to a recent study, more than half of liver donors positive for anti-HBc had detectable cccDNA in liver specimens. Additionally, an anti-HBc IgG titer >4.4 COI (cut-off index) was proposed as a value confirming cccDNA persistence [76]. However, a low titer of anti-HBc may have a high possibility of false-positive or false-negative results [63]. The incidence of false-positive results of anti-HBc ranges from above 10% to more than 50% [70,88,89,90]. These differences reflect from the differences in biological assays that were used and should be verified by the use of a more specific method. Although radioimmunoassay has been shown to be more specific in comparison to commonly used enzyme immunoassays showing 26% false-positive results, it is still not available in clinical practice [91]. Nevertheless, the combination of anti-HBc and anti-HBs levels was recently suggested to be useful for predicting the development of HBV reactivation. In a retrospective study of 197 patients with lymphoma and resolved HBV infection baseline anti-HBc/anti-HBs levels were proposed as helpful in identifying patients at high risk of infection reactivation [75]. Interestingly, another study reported that anti-HBs-negative patients with high anti-HBc titers experienced a significantly higher rate of HBV reactivation than patients with both low anti-HBc and high anti-HBs levels [92]. However, still, a lot of questions exist regarding the importance of anti-HBs and anti-HBc antibody titers in the prediction of HBV reactivation. First of all, the incidence of false-positive anti-HBc results should be ruled out to clarify whether the quantification of anti-HBc antibodies could be used to predict HBV reactivation before the appearance of anti-HBs. Moreover, large cohort studies regarding changes in anti-HBc and anti-HBs levels over time and their influence on the outcomes should be analyzed to evaluate their prognostic and/or diagnostic potential.

### 3.4. Potential Clinical Applications

Summarizing, a series of new biomarkers of virus activity has been identified and correlated with different phases of HBV infection (Table 1). Their application in clinics could increase understanding of HBV pathogenesis and patient disease progression and response to treatment. Looking in the future, cccDNA is not likely to become a routine biomarker because it requires liver biopsy. Nevertheless, both the level and transcriptional activity of cccDNA are extremely important for evaluating novel treatment strategies. A deep review of the existing literature led us to the conclusion that both serum HBcrAg and HBV RNA would be most valuable in clinical practice. As both markers reflect the amount and replication activity of intrahepatic cccDNA, they could be useful for evaluating the efficiency of novel direct-acting agents that target cccDNA and the ability to achieve a true cure. Their use in clinical practice could be reasonably useful for diagnosis, monitoring, and prognostication in patients with CHB. Serum HBcrAg and HBV RNA levels are significantly correlated, and their decreasing ratio is similar during NA therapy. They might be, therefore, useful biomarkers for monitoring treatment response, or for identification of patients who may successfully discontinue NA therapy. Moreover, serum HBcrAg is now intensively studied as a potential predictor for hepatocellular development, and the risk of HBV reactivation after immunosuppressive therapy. Serum HBV RNA, particularly pgRNA, reflects viral replication activity and should be most valuable for monitoring the effect in patients receiving novel anti-HBV therapies. These viral molecules are promising as surrogate markers for HBV viral activity, and in combination with standard biomarkers will allow for better assessment of HBV infection.

## 4. Artificial Intelligence for the Diagnosis of Hepatitis B

Hepatitis B diagnosis and management are challenging due to the broad range of diagnostic markers including viral, host, and liver disease factors. Beyond well-known diagnostic tools, large progress has been made in discovering new viral and host diagnostic factors with a high potential for supporting treatment decisions, predicting outcomes, or the susceptibility to infection. Such a huge amount of data is hard to process and may sometimes cause difficulty in making a decision or at least delay it. Currently, it is possible to streamline the diagnostic process and avoid misdiagnosis by the use of artificial intelligence (AI) methods. AI may be defined as any computer system that can handle diverse types of data and analyze them as it is observed by the human brain. In other words, it is a simulation of human intelligence performing functions associated with the human brain, such as learning and problem-solving. AI programs are trained based on interpretations and decisions made by physicians before the implementation of the AI system so that they can accurately deal with similar health data. As a branch of computer science, the field of AI mainly focuses on machine learning (ML) and deep learning (DL). Still, it is difficult to distinguish these terms as they are highly associated. Nevertheless, they are not interchangeable. AI is a field focused on automating intellectual tasks normally performed by humans, and ML and DL are specific methods of achieving this goal [93]. As a subset of AI, ML provides systems the ability to automatically learn and improve the performance of a specific task. DL is a machine learning technique that creates networks capable of learning in an unsupervised manner from unstructured and unlabeled data. Finally, artificial neural networks (ANNs) are the most popular machine learning algorithms to date, inspired by the neuroanatomy of the brain [94].

The use of machine learning algorithms to predict disease risk has gained the attention of the healthcare field and biomedical research. Moreover, these algorithms have been successfully applied for the diagnosis and management of viral hepatitis. Based on machine learning, a predictive model for inflammation grades of chronic hepatitis B was proposed. This large-scale study analyzed HBV-infected samples by combining gene expressions data and three clinical parameters (ALT, AST, HBV DNA) [95]. Furthermore, the user-friendly web tool (LiveBoost) was applied for the clinical prediction of HBV and HCV related hepatic fibrosis. In this study, an original FIB-4 score (non-invasive fibrosis scoring systems) was compared with machine learning methods. In consequence, the ML system outperformed FIB-4 scoring in predicting advanced hepatic fibrosis [96]. Additionally, an ANN model was demonstrated as effective in diagnosing liver fibrosis reversion for chronic HBV-induced liver fibrosis patients [97]. Machine learning algorithms have been recently applied for the accurate identification of individuals at high risk of HBV infection and the developed model was proposed as an improvement for the detection rate of positive HBsAg [98]. Furthermore, the ability of ANN to accurately predict HBsAg seroclearance in HBeAg-negative CHB patients was recently demonstrated. Zheng et al. [99] have shown that the new ANN model is superior to the conventional statistical linear approach because it could predict the outcome in a shorter time. Another study has confirmed that machine learning algorithms have appropriate performance in predicting HBsAg seroclearance [100]. An ANN model was also proposed for the treatment prediction in HBV carriers. Based on supervised learning of 90 patient samples it was able to predict 92% of successful therapies [101]. Moreover, Hou et al. [102] have established ANN-based models for predicting 28- and 90-day mortality of HBV acute-on-chronic liver failure. The constructed scoring system was compared with four others related to clinical prognosis, and the superiority of ANN was clearly demonstrated. A machine learning approach turned out to be useful for determining the viral variants that accurately classify HBeAg status as well as for viral genome classification [103,104]. Additionally, the power of artificial intelligence was successfully utilized for predicting the interactions between viral and host proteins, examining the key genes and pathways in hepatocellular carcinoma development as well as for designing new antiviral compounds [105,106].

Over recent years, the range of artificial intelligence applications has been largely increased and it is gradually changing medical practice. There are already many research studies suggesting that AI can be helpful in the diagnosis and management of hepatitis B virus infection. Besides, as clinical AI systems mature there is no turning back from the implementation of machine learning systems in treatment and diagnosis of CHB. Nevertheless, the integration of AI into routine clinical practice seems to be challenging. Firstly, development of AI algorithms will require a laborious process of acquiring, labeling, and preparing training data from numerous healthcare organizations. Once the data will be accessible to AI developers, they can contain strings, international classification of disease codes, or domestic procedural terminology codes. Before the deployment, AI algorithms must perfectly match the local population and local practice patterns. Secondly, implementing an AI algorithm to the clinical practice is limited by the black box phenomenon that is unable to understand how the system creates the prediction and what factors are taken into consideration. Therefore, the physician needs to be familiar with the inputs and algorithms to correctly interpret the AI-proposed diagnosis, and ensure that proposed algorithms could improve patient care. Finally, it is also important to consider ethical challenges. Several of these concern the privacy of medical and patient data, or psychological harm when patients learn that they will likely suffer for serious malignancies or mental disorders in later life. Another problem is that multiple factors may affect AI predictions, and the usage of data containing mistakes creates incorrect algorithm output. Moreover, AI calculations do not include features that are basal for decision making in clinical practice. These include, based on ethical principles, non-maleficence, beneficence, respect for patient autonomy, and justice. Clearly, there is much work to do in order to translate AI research into clinically validated systems. However, physicians and machines working in combination seem to display the greatest potential to improve clinical decision making and patient health outcomes. Nevertheless, before their adoption in clinical practice, AI algorithms must be critically reviewed and validated by the use of large amounts of high-quality training data. Moreover, adequately designed interdisciplinary studies with frontline clinicians are crucial to ensure patients’ benefit and safety as well as to avoid any unexpected harm.

## 5. New Direct-Acting Antivirals for HBV

The main challenge in the management of CHB patients is the lack of efficient treatment strategies that could achieve HBV elimination. Current antivirals offer at best control of infection improving patient survival and quality of life by preventing disease progression, and consequently HCC development. However, none of the licensed therapeutics can reliably achieve a virological cure as they have little effect on a unique intracellular viral replication intermediate (cccDNA) which ensures progeny virus production. Only a few cccDNA copies per hepatocyte can reactivate full virus production upon therapy withdrawal or loss of immunological control of the low-level replication going on even under therapy. At present, there are two treatment strategies approved for the treatment of adults with CHB which include interferon-α (Peg-IFNα) and nucleos(t)ide analogs (NAs): nucleosides (lamivudine, telbivudine, entecavir) and nucleotides (adefovir and tenofovir). NAs are safe, generally well-tolerated, and can suppress viral replication to undetectable levels. This may reduce the risk of disease progression, and can result in liver fibrosis and cirrhosis regression. However, a long-term treatment, which is required to maintain virological control, often leads to the selection of drug-resistant mutants harboring amino acid substitutions in the viral polymerase or provokes serious side effects (nephrotoxicity, osteopenia, bone marrow aplasia, etc.). Moreover, HBsAg loss is rarely achieved by NAs. The other approved therapy, Peg-IFNα, despite its finite duration and higher probability to achieve HBsAg clearance, is limited by high costs, numerous side effects, and low response rate [107,108]. The combination of Peg-IFNα and NAs increases the rate of a functional cure, but it is limited to a relatively small proportion of patients [109]. Therefore, there is an urgency for novel therapeutic drugs that could achieve a sustained loss of HBsAg and reduce the viral reservoir cccDNA by targeting different steps of the HBV replication cycle or modulating host immune response. Several novel therapeutic strategies are under investigation that directly target HBV, which include entry inhibitors, drugs and gene editing tools targeting cccDNA, capsid assembly modulators, RNA interference compounds, HBsAg secretion inhibitors, and ribonuclease H inhibitors.

### 5.1. HBV Entry Inhibitors

The recent discovery that NTCP is a functional receptor for HBV entry into hepatocytes, has opened the door for the potential therapeutic application of entry inhibitors for protecting uninfected hepatocytes. First, in class agent, bulevirtide (Myrcludex-B, Myr-B), is a myristoylated synthetic lipopeptide derived from the preS1 domain of the HBV envelope (Table 2). Binding to NTCP, it effectively prevents HBV spreading and inhibits amplification of intrahepatic cccDNA [110]. The first in-human application of Myr-B showed excellent tolerability up to high doses suggesting that no serious off-target effects are expected [111]. Phase II of a clinical trial showed that higher doses of Myr-B had more antiviral potency, but no influence on HBsAg level was observed [109]. Additionally, in phase III Myr-B exposed comparable efficacy but a better histological response in comparison to tenofovir. Moreover, it was suggested that Myr-B treatment is efficacious and safe for long-term use in treatment-naïve and tenofovir-experienced patients with CHB [112].

Cyclosporin A (CsA) is a well-known immunosuppressive agent that is widely used for the prevention of rejection after organ transplantation. Few studies have proposed CsA as a potential inhibitor of HBV entry due to its ability to target an NTCP transporter activity [113,114,115]. However, recent findings have suggested that CsA and its derivatives may inhibit the function of NTCP for the uptake of bile acids into hepatocytes. Therefore, attention has been drawn on new compounds with the ability to inhibit HBV entry without significant loss of NTCP transporter function. Interestingly, Shimura et al. [116] identified two small-molecules CsA analogs, SCY450 and SCY995, which did not impair the NTCP-dependent uptake of bile acids and could inhibit multiple HBV genotypes including drug-resistant HBV variants. Another compound, cyclosporin inhibitor CRV431, was found to reduce liver HBV DNA and HBsAg levels in transgenic mice [117]. The results of the first phase of clinical trials showed that this drug is safe and well-tolerated at several doses [118].

### 5.2. cccDNA Formation Inhibitors/Inactivators

As none of the available drugs can target cccDNA, new molecules that are able to inhibit cccDNA formation or at least destabilize the already existing pool are currently being explored (Table 2). Recent advances have been made in developing new strategies and several options are now available for cccDNA targeting, to abolish HBV persistence. Disubstituted sulfonamides (DSS) were the first compounds identified as inhibitors of cccDNA production in 2012. Structurally related CCC-0975 and CCC-0346 were able to disrupt the formation of cccDNA from rcDNA [132]. Furthermore, interferon-mediated APOBEC3 upregulation was proposed as a strategy to eliminate cccDNA. It was shown that IFN-α in addition to lymphotoxin-β receptor (LTβR) activation induces cccDNA degradation through induction of nuclear APOBEC3 deaminases without hepatotoxicity [133,134]. Nevertheless, Meier et al. [135] have demonstrated that although the lymphotoxin pathway was activated during chronic HBV infection, LTβR and APOBEC3 gene expression was not correlated with intrahepatic cccDNA levels. They concluded that activation of the LTβR/APOBEC3 pathway has no major impact on cccDNA.

Currently, the two most commonly used genome editing technologies that target and cleave cccDNA are transcription activator-like endonucleases (TALENs), and clustered regularly interspaced short palindromic repeats (CRISPR)-associated system 9 (Cas9) proteins [136]. The first study regarding the efficacy of TALENs to disrupt hepatitis B replication was published in 2013. Approximately 31% reduction of cccDNA in HepG2.2.15 cells were reported without evident toxicity. Inhibition of viral replication markers was also observed in vivo in a murine model [137]. Another investigation has demonstrated that the expression of TALENs reduces the production of pgRNA, viral proteins, and the amounts of cccDNA [119]. The use of the CRISPR/Cas9 system was first reported in 2014, indicating its potential in eradicating persistent HBV infection without apparent toxicity [120]. Several other studies have demonstrated successful inhibition of virus replication and the production of viral markers when employing CRISPR/Cas technologies [121,122]. It was also shown that adeno-associated virus (AAV) delivered CRISPR-SaCas9 could efficiently inhibit serum HBsAg and HBeAg in HBV transgenic mice [138]. Additionally, Kostyushev et al. [123] assessed the anti-HBV activity of CRISPR/Cas9 and cccDNA repair outcomes in an altered NHEJ/HR environment demonstrating that inhibiting NHEJ reduces cccDNA degradation. They concluded that CRISPR/Cas9 systems have high efficiency in cleaving episomal HBV cccDNA and the integrated HBV genome.

### 5.3. Capsid Assembly Inhibitors/Modulators

The HBV core protein, the structural component of the viral nucleocapsid, has recently emerged as a suitable target for antiviral drugs due to its multiple roles in different steps of the HBV replication cycle including genome packaging, reverse transcription, intracellular trafficking, and potential for modulation of cccDNA. Since the HBV nucleocapsid contains the replication complex, targeting its formation is a viable approach for HBV therapy [34]. So far, several capsid assembly modifiers (CAMs) have been investigated and they may be divided into two categories: heteroarylpyrimidines (HAP) which lead to the formation of aberrant or non-capsid structures and phenylpropenimides (PP) or sulfamoylbenzamide (SBA) with derivatives that form morphologically normal capsids lacking viral nucleic acid [109].

The first HAP compound, BAY 41-4109, was shown to decrease the HBV genome replication and the amount of HBV core protein in stably transfected HepG2.2.15 cells [139]. Furthermore, its antiviral activity was confirmed in transgenic mice in which BAY 41-4109 caused inhibition of HBV replication as well as a reduction of HBcAg [140]. Several other studies confirmed the ability of BAY 41-4109 to reduce HBV replication. Nevertheless, a rapid rebound of HBV viremia at the end of the treatment period was reported [31,141]. Recent studies have shown that a novel compound GLS-4 is less toxic and more potent in decreasing viral replication in cell culture in comparison to BAY 41-4109 [142,143]. GLS-4 is the first HAP molecule that has entered clinical trials being currently in phase II in China (Table 2). The results of the first in-human trial have demonstrated that GLS-4 is well tolerated without serious adverse events (SAEs) or dose-limiting toxicity [124].

Among phenylpropenamide capsid inhibitors, several compounds deserve attention. NVR 3-778 is now in clinical phase II trials in combination with other antiviral agents. The preclinical studies have demonstrated its high antiviral activity in HepG2.2.15 cells as well as in a HBV humanized mouse model [125,126]. In phase I clinical study NVR 3-778 was well tolerated and demonstrated antiviral activity with the notably greatest reduction of HBV DNA and RNA for combination treatment with NVR 3-778 and Peg-IFNα [144]. Another SBA compound, JNJ-6379, is a novel and potent inhibitor of HBV replication in vitro across genotypes A to D. Studies have shown that it can inhibit early and late steps of viral replication by preventing the formation of cccDNA and reducing intracellular HBV RNA levels [145]. JNJ-6379 has recently completed the phase I trial and doses up to 600 mg were well-tolerated in healthy Caucasian volunteers. Moreover, JNJ-6379 has demonstrated potent anti-HBV activity by reducing HBV DNA and RNA levels in adult treatment-naìve CHB patients. Based on these the safety profile and plasma exposures in healthy subjects, JNJ-6379 has entered a phase III clinical trial [127]. Lately, a novel potent core protein allosteric modulator ABI-H0731 was investigated and multiple targets of ABI-H0731 in the HBV replication cycle were found. The results of the first phase of clinical trials have shown no serious or severe adverse drug-related events at doses up to 300 mg daily in chronic HBV infection. ABI-H0731 produced promising results also in phase IIa studies when combined with NAs, and provided rapid and enhanced anti-HBV activity [128].

### 5.4. RNA Interference

RNA interference (RNAi) is a natural process in which double-stranded RNA degrades mRNA or blocks translation causing the inhibition of gene expression. As the HBV genome has a compact nature organized in four overlapping reading frames, small interfering RNAs (siRNAs) seem to be the most promising tool in the prevention of HBV infection and the eradication of persistent infection [146]. Several preclinical studies have demonstrated that siRNA has the potential for the inhibition of HBV replication. ARC-520 is a leading siRNA that has already completed phase II study. The first experiments performed on animals models showed that ARC-520 is highly effective at reducing circulating HBV DNA levels as well as serum HBV antigens [147,148]. In phase I trials ARC-520 showed an acceptable tolerability profile with adverse-event frequency the same as placebo. Nevertheless, the authors proposed pretreatment with an oral antihistamine is recommended for future studies [149]. In a phase II trial, ARC-520 was active in NA-experienced HBeAg-positive and HBeAg-negative patients. However, the absolute reduction of HBsAg levels was less efficient in HBeAg-negative patients. Such a limited efficacy was explained by the fact that ARC-520 targets cccDNA-derived pgRNA but not the integrated HBV DNA. This problem has been solved by designing the second generation of siRNAs: ARO-HBV (JNJ-3989) and AB-729 targeting both sources of HBsAg [150]. Preclinical studies have indicated their antiviral activity in all types of patients. Initial phase I/II clinical studies of JNJ-3989 demonstrated that it was safe and well-tolerated with a high ability to reduce all measurable viral products, including HBsAg regardless of HBeAg status. AB-729 is now in phase Ia/Ib to investigate its safety and tolerance on healthy volunteers (Table 2). However, in vitro studies have already presented its potential to reduce HBV RNA, HBeAg, and HBsAg expression [109,129].

### 5.5. HBsAg Secretion Inhibitors

The clearance of HBsAg is one of the goals of a functional cure. As HBV produces HBsAg at a level that far exceeds what is required for virion assembly, its high level in the circulation may contribute to the immunopathogenesis of HBV persistent infection. Thus, there have been efforts to investigate new compounds that can block the release of HBsAg from hepatocytes. Nucleic acid polymers (NAPs) are the newest and most interesting members of antiviral agents that block the release of HBsAg. NAPs are oligonucleotide-based compounds that display broad spectrum antiviral activity in enveloped viruses [109]. Although their exact mode of action is not clear, it has been shown that NAPs can block the assembly of subviral particles, preventing the release of HBsAg from infected hepatocytes [151]. The therapeutic potential of NAPs (REP 2055) to treat chronic HBV infection was assessed on ducks with persistent DHBV infection. Obtained data demonstrated that treatment with the REP 2055 can lead to sustained control of persistent DHBV infection [152]. The clinical impact of REP 2055 monotherapy was assessed in eight naïve HBeAg positive hepatitis B patients in 2016. The majority of patients have achieved reduction or clearance of serum HBsAg and reduction of serum HBV DNA. However long-term treatment with REP 2055 resulted in administration side-effects [153,154]. This tolerability problem has been solved by designing a new NAP compound—REP 2139 (Replicor). Recent results from clinical studies have demonstrated that treatment with new NAPs (REP 2139 and REP 2165) results in high rates of HBsAg clearance in both HBeAg-positive and HBeAg-negative CHB patients. In another study REP 2139 was administered to patients with hepatitis B and hepatitis D virus (HDV) co-infection in combination with Peg-IFNα. This combined treatment was well tolerated and suppressed HBV and HDV in a high proportion of patients after 1 year of therapy [155]. Lately, the results of the study on the safety and efficacy of REP 2139 and REP 2165 in combination with TDF/Peg-IFNα in treatment-naive HBeAg-negative patients were reported. Presented data have shown that 41% of participants achieved functional cure (HBsAg and HBV DNA undetectable) and liver function has normalized in 94% of patients [130].

### 5.6. Ribonuclease H Inhibitors

The HBV DNA polymerase is the only enzyme encoded by the virus. It is a multifunctional protein, which possesses three activities: DNA-dependent DNA polymerase, reverse transcriptase, and RNase H. During the replication cycle, RNase H is responsible for the hydrolyzation of the pgRNA from RNA/DNA hybrids to enable the synthesis of plus-strand DNA. Therefore blocking the activity of the RNase H is an attractive target for new antiviral drugs as it should block the synthesis of viral DNA and the release of mature virions [29,109]. So far, a large number of compounds have been screened against HBV RNase H and several chemical classes have demonstrated the potential. These three chemotypes causing an accumulation of RNA: DNA heteroduplexes in viral capsids include α-hydroxytropolones (α-HT), *N*-hydroxyisoquinolinediones (HID), *N*-hydroxypyridinediones (HPD) [156,157]. RNase H inhibitors have been demonstrated to be not affected by HBV genetic diversity and have presented equal activity regardless of HBV genotype [158]. The efficacy of HPD 208 and the αHT 110 has been analyzed so far in humanized mouse livers. Treatment with both compounds resulted in significant reductions in plasma viremia without affecting HBeAg and HBsAg levels [131]. Several other studies demonstrated the potential of inhibiting the viral ribonuclease H strategy as a promising alternative for developing new anti-HBV drugs. Combinations of RNase H inhibitors with other antiviral agents had additive antiviral effects without enhanced cytotoxicity [159].

## 6. Novel Immunomodulatory Agents for Hepatitis B

The molecular mechanisms of HBV persistence are not fully understood, but it is well known that viral replication itself does not cause liver damage in a short period of time. Therefore, it is accepted that the individual’s immune response is essential for sustained viral control and the inadequate response is associated with chronic hepatitis B [160]. Indeed, 90% of infants whose immune system is not fully matured, become persistently infected exposed to HBV at birth or in perinatal age. In most adults, HBV infection results in self-limited, transient liver disease. However, when innate and adaptive immune response fails adults develop chronic HBV carriage and are at risk of developing chronic hepatitis B and its complications [161]. Although individuals with CHB are not widely immunosuppressed they exhibit a weak anti-HBV T cell response, whereas a broad and multispecific T-cell response is observed in patients who clear acute HBV infection [162]. Therefore, based on the immunopathogenesis of HBV infection, restoring the HBV-specific immunity represents a new strategy for cure in CHB patients. Numerous agents are under development as immunemodulators, which include Toll-like receptor agonists, immune check point inhibitors, engineered T cells, host cellular targets, and therapeutic vaccines.

### 6.1. Toll-Like Receptor Agonists

Toll-like receptors (TLRs) play a vital role in the innate immune system by recognizing conserved pathogen-associated structures called PAMPs, and activating specific gene expression programs and the secretion of inflammatory cytokines and chemokines [163]. The ability of diverse TLRs to inhibit HBV replication in vivo has been presented for the first time by Isogawa et al. [164]. Thereafter, a growing amount of evidence demonstrated that HBV can modulate the expression of TLRs and/or inhibit TLR signaling pathways suggesting that they may be used by the virus to evade host immune responses [165]. Recent findings suggested that TLR7 and TLR8 on trophoblastic cells play an important role in the prevention of intrauterine HBV transmission by inhibiting HBV translocation across trophoblast [166]. The use of TLR7-ligand (GS-9620) has been successfully applied to animal models of HBV infection [167,168]. The administration of GS-9620 to mammals has led to serum and liver HBV DNA suppression, as well as the loss of HBsAg [168]. However, it had no significant effect on HBsAg levels in humans after 12-week administration [169]. Correspondingly, in phase II of clinical study, GS-9620 was safe and well-tolerated in patients with CHB. Nevertheless, no significant declines in hepatitis B surface antigen were observed [170]. Another two TLR7 agonists, RO7020531 and JNJ-4964, which are currently in clinical development, have shown to stimulate immune response in both healthy volunteers and patients with CHB [171]. RO7020531, when combined with a capsid assembly modulator, RO7049389, caused a significant reduction in HBV DNA and HBsAg in a mouse model [109]. Moreover, a recent study reported that RO7020531 was safe and well-tolerated in healthy Chinese volunteers [172].

Selgantolimod (GS-9688) is an orally active, potent, and selective toll-like receptor 8 (TLR8) agonist in clinical development for the treatment of chronic hepatitis B. It was shown to induce a sustained antiviral response in the woodchuck model of CHB [173,174], and was well-tolerated in CHB patients in a randomized blind placebo-controlled study [109]. Currently, GS-9688 is undergoing phase II clinical trials.

### 6.2. Immune Check Point Inhibitors

Exhaustion of adaptive immune response is the major cause of HBV chronicity, as it is essential for obtaining successful control of viral infection. In the course of chronic HBV infection, T cells have a weak and dysfunctional response against HBV due to the overexpressed inhibitory receptors including programmed cell death 1 (PD-1), cytotoxic T-lymphocyte associated protein 4 (CTLA-4), or T cell immunoglobulin and mucin protein 3 (TIM-3) [175]. Blocking these immune checkpoints may induce some improvement by promoting the proliferation of HBV-specific T cells and restoring the function of exhausted T cells [176]. The utility of the PD-1/PD-L1 pathway to successfully enhance virus-specific T cells was demonstrated in HBV mouse and woodchuck models [177,178]. However, the PD-1 blockade alone showed only limited ability to restore the immune functions in HBV infection [179,180] and in further studies it was evaluated in a combination with inhibitors of other regulatory pathways [181]. Recently, a synergistic effect of OX40 stimulation combined with PD-L1 blockade was shown to functionally augment HBV-specific CD4 T cells, with the increased secretion of IFN-γ and IL-21 in vitro [182]. Moreover, in vivo blockade of the PD-1/PD-L1 pathway in combination with nucleoside analogue treatment and therapeutic DNA vaccine, induced a strong antiviral effect, achieving a complete viral clearance in treated woodchucks [178]. Additionally, improved infection control was observed in woodchucks after combination therapy with inhibition of PD-L1 and entecavir (ETV) in comparison to ETV treatment alone [183]. Lately, PD-1 inhibitor (nivolumab), was investigated in a phase Ib of clinical study with or without HBV therapeutic vaccine (GS-4774), Nivolumab was well-tolerated in HBeAg negative CHB infected patients, but no significant decline was observed in HBsAg in patients receiving nivolumab alone [184].

### 6.3. Engineered HBV Specific T Cells

The significance of HBV-specific T cell reconstitution in the control of infection has driven the development of strategies based on adoptive transfer of engineered HBV-specific T cells. Current T-cell-based therapies against HBV utilize T cells engineered with a chimeric antigen receptor (CAR) specific for HBsAg, and a classical T cell receptor (TCR) specific for HLA-class I restricted HBV epitopes [185]. The basic principle of this process is to improve the low or depleted energy of a patient’s T-cells by introducing a new well-functioning T-cell library [186]. It should therefore allow for immunological control of HBV infection and in consequence a CHB cure in the long term. For example, patients with leukemia and chronic HBV infection after bone marrow transplantation from vaccinated subjects or individuals with spontaneously resolved HBV infection displayed protective HBs antibodies and cleared HBV infection [187,188]. Interestingly, patients with past HBV infection experienced a spontaneous anti-HBs re-seroconversion after liver transplantation from an HBV-infected donor [189]. Furthermore, in vitro studies have been assessed in HBV transgenic mice and in patients with relapses of HBV-related HCC [190,191,192]. T cells with a CAR specific for HBV envelope proteins rapidly and efficiently controlled HBV replication in HBV transgenic mice, causing only transient liver damage [190]. Evaluating HBsAg-CAR T cells in HBV infected human liver chimeric mice resulted in a decrease of serum HBsAg, viral load, and the amount of HBcAg-positive hepatocytes [193]. Long lasting antiviral effects were also demonstrated for second-generation CAR T cells [194]. In humans, immunotherapy of HBV-related HCC patients with HBV-specific TCR reprogrammed T cells caused a decrease of HBsAg production by HCC cells [191]. Very recently, Tan et al. applied TCR T cells therapy in two patients with HCC recurrence after liver transplantation without significant adverse events [195]. Although engineered T cells therapy shows promise for immune recovery and elimination of HBV-infected hepatocytes, it remains complicated to apply for a large number of patients. As a very expensive personal therapy, it requires specialized personnel and infrastructure. Nevertheless, HBV-specific CAR/TCR-T cells represent a new approach with great therapeutic potential and further research will allow immunotherapy to be considered a real therapeutic option in chronic HBV infection.

### 6.4. Therapeutic Vaccines

The goal of a therapeutic vaccine is to stimulate or boost HBV-specific B- and T-cells in order to activate antiviral immune responses to remove and/or cure HBV-infected hepatocytes without host cell damage [196] To date, different categories of therapeutic vaccines formulations have been evaluated in both animal models and humans, including protein- or peptide-based, DNA-based, and vector-based vaccines [197,198]. It should be underlined that most vaccines are evaluated in a combination with antiviral drugs and despite promising results in preclinical studies, similar approaches failed in chronically HBV-infected patients [199]. To date, several vaccine candidates have been developed and tested in clinical trials for chronically HBV infected patients [196,197,198]. Some of them are discussed below.

Among therapeutic vaccines belonging to the protein-based category, GS-4774 uses recombinant *Saccharomyces cerevisiae* yeast to express HBV-specific antigens such as HBx protein and large HBsAg [196]. Promisingly, GS-4774 showed immunogenicity in mice and healthy human volunteers without serious adverse effects [200]. However, in a phase II trial in virally suppressed CHB patients, no significant reductions in serum HBsAg, and no case of HBsAg clearance was observed [201]. Another study of CHB patients treated with tenofovir alone or combined with GS-4774 resulted in no reduction of HBsAg levels in patients. However, increased production of IFNG, TNF, and IL2 was observed from individuals administered GS-4774 and tenofovir showing the ability of GS-4774 to stimulate host CD8+ T cell responses [202]. Therefore, GS-4774 might be useful in combination with other antiviral agents to boost the anti-HBV immune response.

The nasal vaccine candidate (NASVAC) contains both HBsAg and HBcAg recombinant proteins [196]. The results of the phase I clinical trial showed that the nasal vaccine candidate was well tolerated, safe, and immunogenic in healthy adults [203]. Further studies in chronic patients confirmed its safety and showed that the vaccine strengthened viral control [204,205]. In a recent phase III study, NASVAC was compared with pegylated interferon, and it presented superior serological response and ability to suppress viral DNA. Additionally, NASVAC was safer and demonstrated a lower progression to cirrhosis fibrosis, when compared with pegylated interferon [206].

HepTcell is another vaccine formed by nine synthetic peptides comprising HBV-specific T-cell epitopes, that allows being used for any of the circulating HBV genotypes [199]. The safety and immunogenicity of HepTcell administered with entecavir or tenofovir was successfully evaluated in a phase I trial [207]. In 2020, a phase II trial has started in an expanded patient cohort with chronic HBV infection [199].

The yeast-derived-immunogenic complex (YIC) is an interesting candidate vaccine due to its unusual strategy [196]. It consists of yeast-derived recombinant HBsAg and human anti-HBs immunoglobulin. Phase I and phase II studies proved the safety and therapeutic efficacy of YIC vaccine [208,209,210]. Nevertheless, in a phase III clinical trial, HBeAg seroconversion rate decreased in comparison to the phase IIb in the YIC group (7.8%), while the HBeAg seroconversion rate increased in the placebo group (12.9%). Strikingly, no difference between the YIC group and placebo group in decreasing HBV DNA and normalization of liver function was observed [211].

## 7. Future Directions

The ideal goal of HBV therapy is to eliminate all forms of HBV replication to reach a complete cure (also termed “sterilizing cure”). Nevertheless, such a scenario seems to be unattainable due to the persistence of cccDNA in the liver, and the ability of HBV DNA to integrate into the host DNA. Therefore, the aim of new HBV drug discovery is to achieve a functional cure, allowing for treatment discontinuation with no risk of virological rebound and liver disease progression. According to the agreement of international experts, the primary endpoint of phase III trials should be HBsAg loss and undetectable HBV DNA 6 months after completion of treatment [212]. The recent progress in the development of novel HBV therapies is aided by the availability of improved cellular and animal infection models. Furthermore, the identification of new surrogate markers associated with increased survival will also help to develop a cure. The most promising new treatment options under development, can be classified into two functional categories: direct-acting antiviral agents (DAAs) and immune system modulators. At present, the comparison of drugs with different modes of action is difficult, because they are limited to clinical trials and proof of mechanism and safety studies. Both approaches are being evaluated as monotherapies and/or in combination with other agents, but none of these have yet finished phase III trials. Nevertheless, we believe that the best future strategy against HBV should combine drugs targeting several steps of the HBV replication cycle in combination with immune modulators. Treatment with drugs reducing circulating HBV DNA and viral antigen production, with a simultaneous restoration of functionally efficient adaptive responses could possibly control HBV infection in chronically infected patients. In the near future, the results of phase 2 and phase 3 of the clinical study will help to determine which drug combinations may contribute to a functional cure.

## 8. Conclusions

Causing nearly 900,000 deaths every year, HBV remains a major threat to global public health. More than 290 million people worldwide are chronically infected with HBV and even though a prophylactic vaccine and effective antiviral therapies are available, current treatment offers control but not a cure. Over the past decade, considerable progress has been made in hepatitis B control with constant improvements in molecular diagnostic tools, the implementation of widespread immunization programs, and the development of new antiviral therapies inhibiting viral replication in the long term. The discovery of NTCP as a host entry receptor was a milestone which has opened the door for useful experimental systems to analyze the HBV replication cycle and provided new insight regarding host and viral factors which are significant for HBV infection. Furthermore, new treatment strategies that are under investigation have a high potential to decrease or eliminate cccDNA. Going forward, there is a high possibility that these future therapies might result in resolution of HBV infection which would save millions of people and decrease the financial burden associated with the lifelong treatment of patients with chronic hepatitis B. Winning the battle against HBV will take considerable effort globally but it seems to be possible in the near future.

## Figures and Tables

**Table 1:** Potential clinical application of novel viral biomarkers in chronic hepatitis B infection.

Viral Marker	The Possible Predictive Value	References
HBV RNA	NAs treatment response	[39,40,41]
	NAs cessation	[36,42,43,44]
	Peg-IFNα treatment response	[39,43,46,47]
	Functional cure of chronic hepatitis B	[50]
	HBV-induced HCC	[52]
	Intrahepatic HBV cccDNA	[53,54]
HBcrAg	NAs treatment response	[55]
	Peg-IFNα treatment response	[56,57]
	HBeAg/HBsAg spontaneous seroconversion	[58,59,60,61,62]
	HBV reactivation due to immunosuppressive drug treatments.	[63]
	HBV-induced HCC	[64,65,66,67]
	Intrahepatic HBV cccDNA	[68,69]
Anti-HBs	HBV reactivation due to immunosuppressive drug treatments	[70,71,72,73,74,75]
Anti-HBc	HBV reactivation due to immunosuppressive drug treatments	[75,76]
	HBV occult infection	[77]

HBcrAg—hepatitis B core-related antygen; anti-HBs—hepatitis B surface antibody; anti-HBc—hepatitis B core antibody.

**Table 2:** The most important direct-acting antivirals for HBV under development.

Category	Compound	Development Status	Reference
Entry inhibitors	Myrcludex-B	Phase III	[109,112]
	Cyclosporin A	Preclinical	[113,114,115]
	CRV431	Phase I	[118]
cccDNA formation inhibitors/inactivators	TALENs	Preclinical	[119]
	CRISPR/Cas9	Preclinical	[120,121,122,123]
Capsid assembly inhibitors/modulators	BAY 41-4109	Phase I	[109]
	GLS-4	Phase II	[124]
	NVR 3-778	Phase IIa	[125,126]
	JNJ-6379	Phase III	[127]
	ABI-H0731	Phase IIa	[128]
RNA interference	ARC-520	Phase II	[109,129]
	JNJ-3989	Phase I/II	[109,129]
	AB-729	Phase I	[109,129]
HBsAg secretion inhibitors	REP 2139	Phase II	[130]
	REP 2165	Phase II	[130]
Ribonuclease H inhibitors	HPD 208	Preclinical	[131]
	αHT 110	Preclinical	[131]
